# Supplementary material for: Evidence supported by Mendelian randomization: impact on inflammatory factors in knee osteoarthritis
Source: Front Med (Lausanne). 2024 May 28;11:1382836. doi: 10.3389/fmed.2024.1382836 (PMC11165061; doi:10.3389/fmed.2024.1382836)
Supplement: Supplementary file 1 [file Data_Sheet_1.ZIP › data/GCST90274781_5e6/781.leaveoneout.pdf]

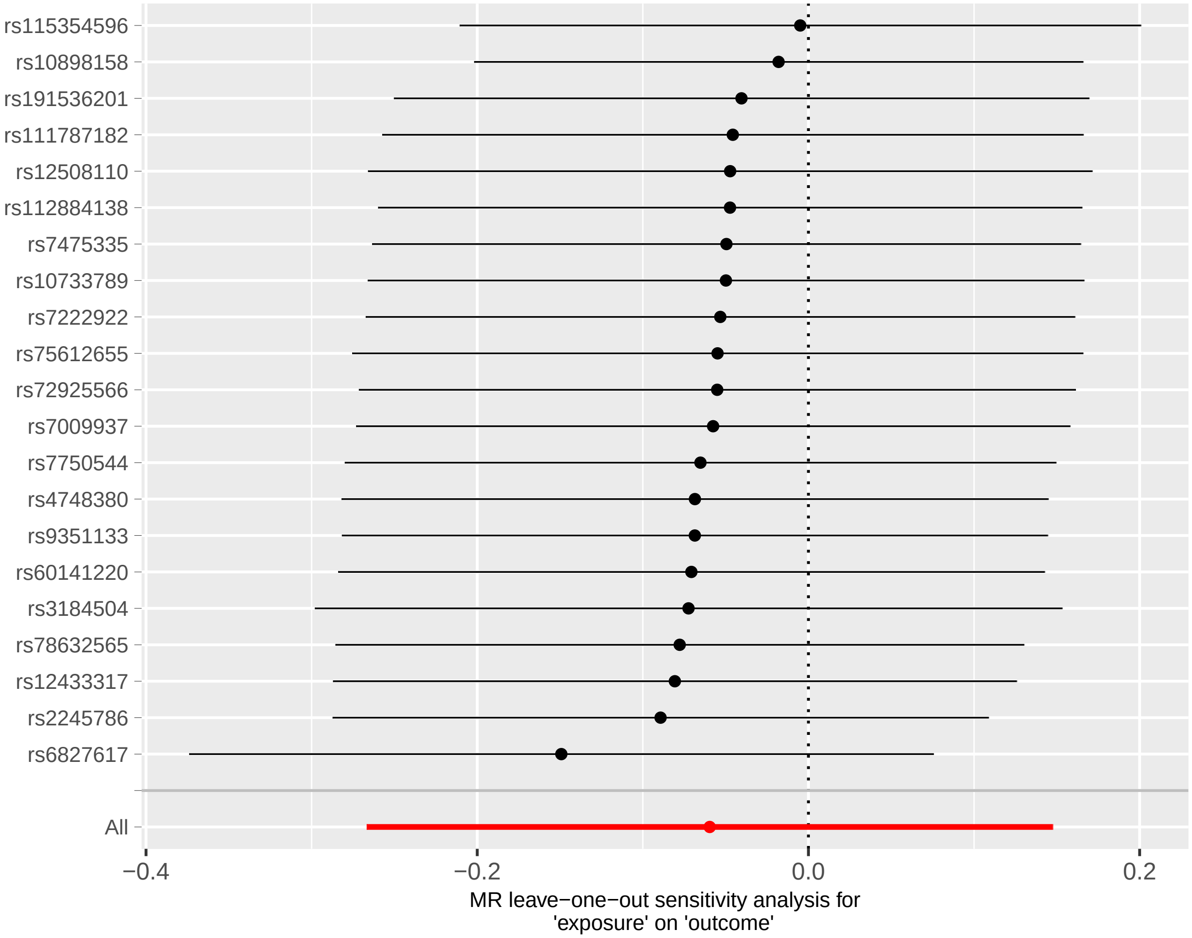

rs115354596
rs10898158
rs191536201
rs111787182
rs12508110
rs112884138
rs7475335
rs10733789
rs7222922
rs75612655
rs72925566
rs7009937
rs7750544
rs4748380
rs9351133
rs60141220
rs3184504
rs78632565
rs12433317
rs2245786
rs6827617
All
−0.4
−0.2
0.0
0.2
MR leave−one−out sensitivity analysis for
'exposure' on 'outcome'
